# Complete Genome Sequences of Bioluminescent Staphylococcus aureus Strains Xen31 and Xen36, Derived from Two Clinical Isolates

**DOI:** 10.1128/mra.00024-23

**Published:** 2023-02-22

**Authors:** Océane Blaise, Clarisse Leseigneur, Thomas Cokelaer, Elena Capuzzo, Nadira Frescaline, Olivier Dussurget

**Affiliations:** a Institut Pasteur, Université Paris Cité, CNRS UMR 6047, Unité de Recherche Yersinia, Paris, France; b Institut de Recherche Biomédicale des Armées, INSERM UMRS-MD 1197, Centre de Transfusion Sanguine des Armées, Clamart, France; c École Polytechnique, Sorbonne Université, Laboratoire de Physique des Plasmas, CNRS, Palaiseau, France; d Institut Pasteur, Université Paris Cité, Plate-forme Technologique Biomics, Paris, France; e Institut Pasteur, Université Paris Cité, Hub de Bioinformatique et Biostatistique, Paris, France; University of Rochester School of Medicine and Dentistry

## Abstract

Here, we report complete genome sequences of two clinical isolates of Staphylococcus aureus, namely, Xen31 and Xen36, which have been genetically modified to express an optimized Photorhabdus luminescens luciferase operon. Xen31 and Xen36 are bioluminescent strains used widely for investigation of bacterial pathogenesis, drug discovery, and development of novel therapies.

## ANNOUNCEMENT

Staphylococcus aureus is a saprophytic bacterium that can infect multiple animal hosts, including humans (1–11). Methicillin-resistant S. aureus (MRSA) infections led to more than 100,000 deaths in 2019 worldwide (12). Thus, the development of innovative therapeutics is urgently needed. Bioluminescent microorganisms are powerful tools to use to investigate pathogenesis, screen antimicrobial drugs, and assess therapies (13–31). Here, we report genome sequences of bioluminescent S. aureus strains Xen31 and Xen36 (Perkin Elmer 119242 and 119243), derived from clinical isolates (16, 32). A modified Photorhabdus luminescens *luxABCDE* operon is integrated in the Xen31 chromosome and Xen36 plasmid. Genomic characterization of these strains is of interest as they are commonly used in infection models and preclinical studies (22, 27, 28, 31, 33–42).

Genomic DNA was isolated from bacteria grown in brain heart infusion broth (BD) using a PureLink DNeasy kit (Invitrogen), sheared in Covaris g-tubes at 5,500 rpm for 60 s twice, and size selected using Pacific Biosciences (PacBio) magnetic beads diluted 3.5-fold. Sequencing was performed using a PacBio Sequel I instrument. Libraries were prepared according to the PacBio protocol for multiplex SMRT sequencing with v3 chemistry using a SMRTbell express template prep kit 2.0 and barcoded adapters kit 8A/8B. Circular consensus sequences (CCSs) were constructed using SMRT Link software v6.2.0 (PacBio) with default settings except that minimum accuracy was set to 0.7. Bioconvert v0.6.2 (https://github.com/bioconvert/bioconvert) converted CCS files to FastQ files resulting in 40,653 and 29,054 CCS reads for Xen31 and Xen36 genomes, respectively. *N*_50_ values were 9,122 and 9,890 bp, respectively. CCS data had high quality with a Phred score above 40 for 95% of reads. Taxonomic analysis using the Sequanamultitax pipeline (43) showed 99.9% Staphylococcus aureus reads. Assemblies were performed using the LORA pipeline from Sequana (43). Default parameters were used for all software. Using the Canu v1.6 assembler (44), four contigs were obtained for Xen31. Circularization was performed with Circlator v1.5.5 (45). The main circularized contig was 2,966,802 bp long with a G+C content of 32.95% (Table 1) and exhibited uniform coverage of 98×. The second circularized 32,188-bp contig had a uniform 105× coverage and was identified as plasmid CP041009.1 by BLAST. The 43,128-bp-long third contig had a uniform coverage of 500×. It was identified as phage ECel-2020n (CP062457.1) by BLAST and phage AJ_2017 (NC_048644) by the PHASTER Web server (46). The fourth contig of poor coverage was discarded. Analysis of the Xen36 sequence with Canu v1.6 and Flye v2.9 (47) resulted in a main contig of 2,749,617 bp with 80× coverage, 32.91% G+C content (Table 1), and 4 small contigs, identifying 28,816-bp plasmid CP082814 and 13,162-bp plasmid CP029650.1. Phages of the Xen31 and Xen36 main contigs were detected by PHASTER (Table 1).

**TABLE 1:** General features of the Staphylococcus aureus strains Xen31 and Xen36^*a*^

Feature	Data by strain	
	Xen31	Xen36
Strain origin	Clinical isolate	Clinical isolate
Resistance type	MRSA	MSSA
Sequence type	ST247	ST8037
Genome size (bp)	2,966,802	2,749,617
G+C content (%)	32.95	32.91
No. of protein-coding genes	2,742	2,528
No. of rRNAs	19	19
No. of tRNAs	62	60
No. of tmRNAs	1	1
No. of CRISPR regions	5	10
Prophage (accession no.)	PT1028 (AY954948.1), ϕNM3 (NC_008617), ϕJB (NC_028669), AJ_2017 (NC_048644)	PT1028 (AY954948.1)
Plasmid accession no.	CP041009.1	CP082814 and CP029650.1
Antibiotic resistance genes	*aac(6′)-aph(2′′)*, *aadD*, *ant(9)*-Ia, *bla*TEM-1A, *bleO*, *cat*(pC194), *erm*(A), *erm*(B), *fosB*, *mecA*, *tet*(M)	*aph(3′)*-III and *blaZ*
Virulence determinant genes^*b*^	*adsA*, *agr*, *ahpCF*, *arlRS*, *aur*, *cap*, *chp*, *clf*, *coa*, *codY*, *crt*, *dlt*, *eap*, *ebp*, *efb*, *esa*, *ess*, *esx*, *flr*, *fnb*, *fur*, *geh*, *graRSX*, *hla*, *hld*, *hlgABC*, *hysA*, *ica*, *isd*, *katA*, lukED*, *lukGH*, *lytRS*, *mgrA*, *mprF*, *oatA*, *psm*, *RNAIII*, *rot*, *rsr*, *saeRS*, *sak*, *sar*, sarT*, sarU*, *saxG*, *sbi*, *scn*, *sdr*, *sea*, *see*, selP*, set1*, set3*, set5*, set6*, set9*, set12*, set21*, set30–34*, set36–40*, *set2*, *set4*, *set7*, *set8*, *sigB*, *sodA*, *sodM*, *spa*, spl*, *spn*, *srrAB*, *ssl*, *ssp*	*adsA*, *agr*, *ahpCF*, *arlRS*, *aur*, *cap*, *chp*, *clf*, cna*, *coa*, *codY*, *crt, dlt*, *eap*, *ebp*, *efb*, *esa*, *ess*, *esx*, *flr*, *fnb*, *fur*, *geh*, *graRSX*, *hla*, *hld*, *hlgABC*, *hysA*, *ica*, *isd*, *katA*, *lukGH*, *lytRS*, *mgrA*, *mprF*, *oatA*, *psm*, *RNAIII*, *rot*, *rsr*, *saeRS*, *sak*, *sar*, *saxG*, *sbi*, *scn*, *sdr*, *sea*, seb*, sec*, *see*, seg*, sei*, selm*, seln*, selo*, selu*, *set2*, *set4*, *set7*, *set8*, set11*, set13*, set15*, set16*, set22*, *sigB, sodA*, *sodM*, *spa*, *spn*, *srrAB*, *ssl*, *ssp*, yent1,2*

a tmRNAs, transfer-messenger RNAs; MSSA, methicillin-susceptible Staphylococcus aureus.

b *, Strain-specific genes.

A total of 2,742 and 2,528 protein-coding sequences were identified in Xen31 and Xen36, respectively (Table 1). Sequence types were defined using PubMLST. Putative clustered regularly interspaced short palindromic repeat sequences were identified by CRISPRCasFinder (48). Annotation was performed with Prokka v1.14.5 (49). Antimicrobial resistance genes were detected using ResFinder-4.1 (50) and GenBank. Virulence genes were identified using Virulence Factor of Pathogenic Bacteria Database (VFDB) and GenBank.

### Data availability.

Sequences were deposited in ENA under the accession number E-MTAB-12210. Raw data, assemblies, and annotations can be accessed online at https://doi.org/10.5281/zenodo.7517362.
